# Neonatal Arnold–Chiari II Malformation: An Imaging‐Focused Case Report

**DOI:** 10.1002/ccr3.71971

**Published:** 2026-01-29

**Authors:** Mohammad Alashqar, Seba Lubbadeh, Ahmad Daraghmeh, Ahmad Alashqar, Zaina Khaled, Suleiman Sbeih, Amjad Bdair, Israa Salman, Mohammed I. Abu Kamesh

**Affiliations:** ^1^ Department of Medicine, Faculty of Medicine and Health Sciences An‐Najah National University Nablus Palestine; ^2^ Department of Medicine, Faculty of Medicine and Health Sciences Alexandria University Alexandria Egypt; ^3^ Faculty of Medicine Al‐Quds University Abu Dis Palestine; ^4^ Pediatrics and Neonatology Tulkarim Governmental Hospital Tulkarim Palestine; ^5^ Radiology Department An‐Najah National University Hospital Nablus Palestine

**Keywords:** Arnold–Chiari malformation type II, hydrocephalus, magnetic resonance imaging, myelomeningocele, neonate, neuroimaging

## Abstract

Arnold‐Chiari Malformation Type II (CM‐II) is a serious congenital hindbrain disorder marked by the displacement of the cerebellum and brainstem downwards through the foramen magnum. CM‐II is frequently linked with myelomeningocele and hydrocephalus. We present a case of a male neonate delivered through C‐section with myelomeningocele, hydrocephalus, and paralysis of the lower limbs. MRI revealed the presence of Arnold‐Chiari Malformation Type II along with dysgenesis of the corpus callosum, absent septum pellucidum, scaphocephaly, and the presence of a small syrinx. Surgical management, including ventriculoperitoneal (VP) shunt placement, was successful. This case underlines the significance of MRI in diagnosis and the necessity of the early multidisciplinary management for the improvement of neonatal outcomes.

## Key Clinical Message

Magnetic resonance imaging (MRI) plays a central role in the early diagnosis of Arnold–Chiari II malformation by delineating hindbrain herniation and associated anomalies. Early surgical intervention and coordinated multidisciplinary care are crucial to minimize complications and improve survival and neurodevelopmental outcomes in affected neonates.

**Abbreviations**
ACM‐II: Arnold–Chiari malformation type II
AP: anteroposterior
CM‐II: Chiari malformation type II
CSF: cerebrospinal fluid
MRI: magnetic resonance imaging
NICU: neonatal intensive care unit
STIR: short tau inversion recovery
SWI: susceptibility‐weighted imaging
VP: ventriculoperitoneal

## 1 Introduction

Arnold‐Chiari malformations are a group of congenital anomalies of the hindbrain structure, predominantly the cerebellum, brainstem, base of skull, and upper cervical cord of the spine [1]. These malformations were established, for the first time, at the end of the nineteenth century when Hans Chiari and Julius Arnold presented a list of structural deformities due to developmental failure of the posterior fossa [2]. Currently, there are four main types of Chiari malformations, categorized based on the extent of herniation of the brainstem and cerebellar structure across the foramen magnum, along with corresponding spinal or cranial abnormalities [3]. In the neonatal period, Chiari type II represents the most severe and clinically significant subtype [4]. It is almost invariably associated with a myelomeningocele, an open spinal dysraphism, and is characterized by the downward displacement of the cerebellar vermis, medulla, and fourth ventricle into the cervical spinal canal [5]. Hydrocephalus, syringomyelia, and tethered cord syndrome commonly accompany this malformation [6]. The above abnormalities cause broad neurological manifestations ranging from motor weakness, feeding and respiratory difficulties, cranial nerve dysfunction, to delayed milestones [7]. The exact etiology of Chiari II malformation remains unknown [4]. The most generally accepted explanation, or the “unified theory”, states that the defective closing of the neural tube in early embryogenesis causes cerebrospinal fluid (CSF) leaking, which causes the small posterior fossa and gradual downward displacement of hindbrain structures [8]. Epidemiologically, the Chiari II malformation is uncommon, with an approximated incidence of about 0.44 per 1000 live births [6]. It affects both genders equally and is more prevalent in the regions of lower maternal folate intake [6]. Periconceptional folic acid supplementation has been shown to significantly reduce the incidence of Chiari II malformation [6]. This case report aims to highlight the role of medical imaging in the diagnosis of Arnold–Chiari malformation type II in a neonate.

## 2 Case History/Examination

A male neonate was delivered via cesarean section due to breech presentation, with a birth weight of 3100 g and Apgar scores of 4 and 6 at one and 5 min, respectively. He required admission to the neonatal intensive care unit (NICU) for 45 days and was exclusively breastfed during his stay. The mother was gravida 3 para 2, had not taken folic acid before or during pregnancy, and had not undergone prenatal ultrasound screening. Her pregnancy and delivery were otherwise uneventful. She was blood type O+, and no significant maternal medical or family history was reported.

The newborn's head circumference and length were recorded as 44 cm and 47 cm, respectively, during the first evaluation. The tone and reflexes of the upper limbs were normal, while in the lower limbs there was no tone, power, or reflexes. The Moro and sucking reflexes were normal. The anterior fontanelle was bulging. A round lumbosacral defect measuring approximately 3 × 2 cm with ruptured overlying skin and no evidence of CSF leakage was observed, consistent with myelomeningocele. The chest and abdomen were normal on examination, and no dysmorphic features were observed.

## 3 Differential Diagnosis, Investigations and Treatment

**FIGURE 1:**
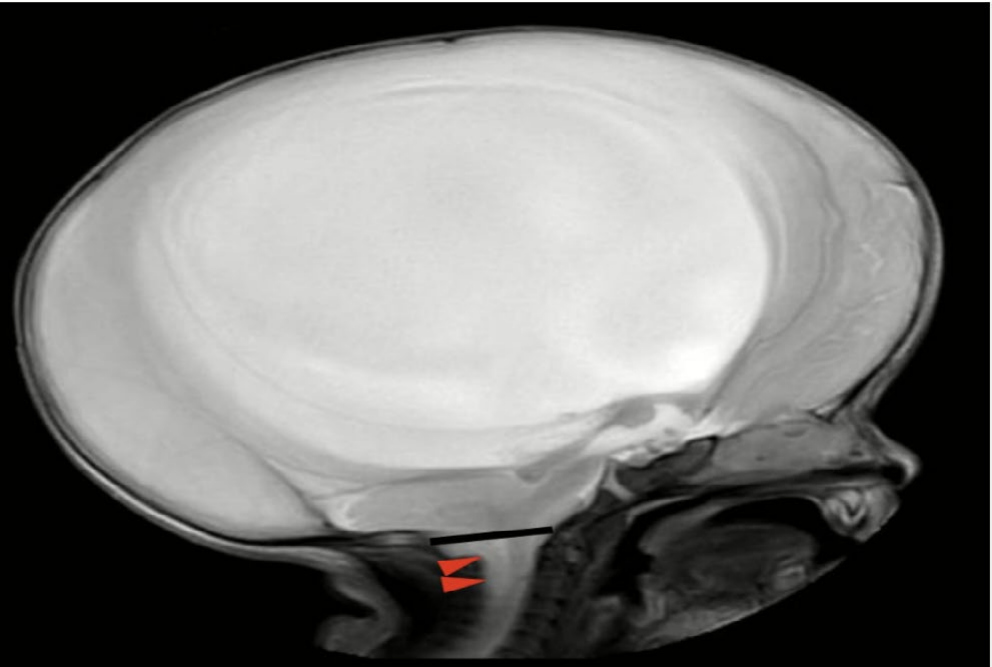
Representative sagittal T2‐weighted MRI of the brain shows a small, crowded posterior fossa with descent of the cerebellar tonsils (red arrowheads) below the basion–opisthion line (black line).

Brain ultrasound showed severe hydrocephalus and thinning of the cortex, prompting further neuroimaging. MRI confirmed the diagnosis of Arnold–Chiari Malformation Type II (Figure 1).

**FIGURE 2:**
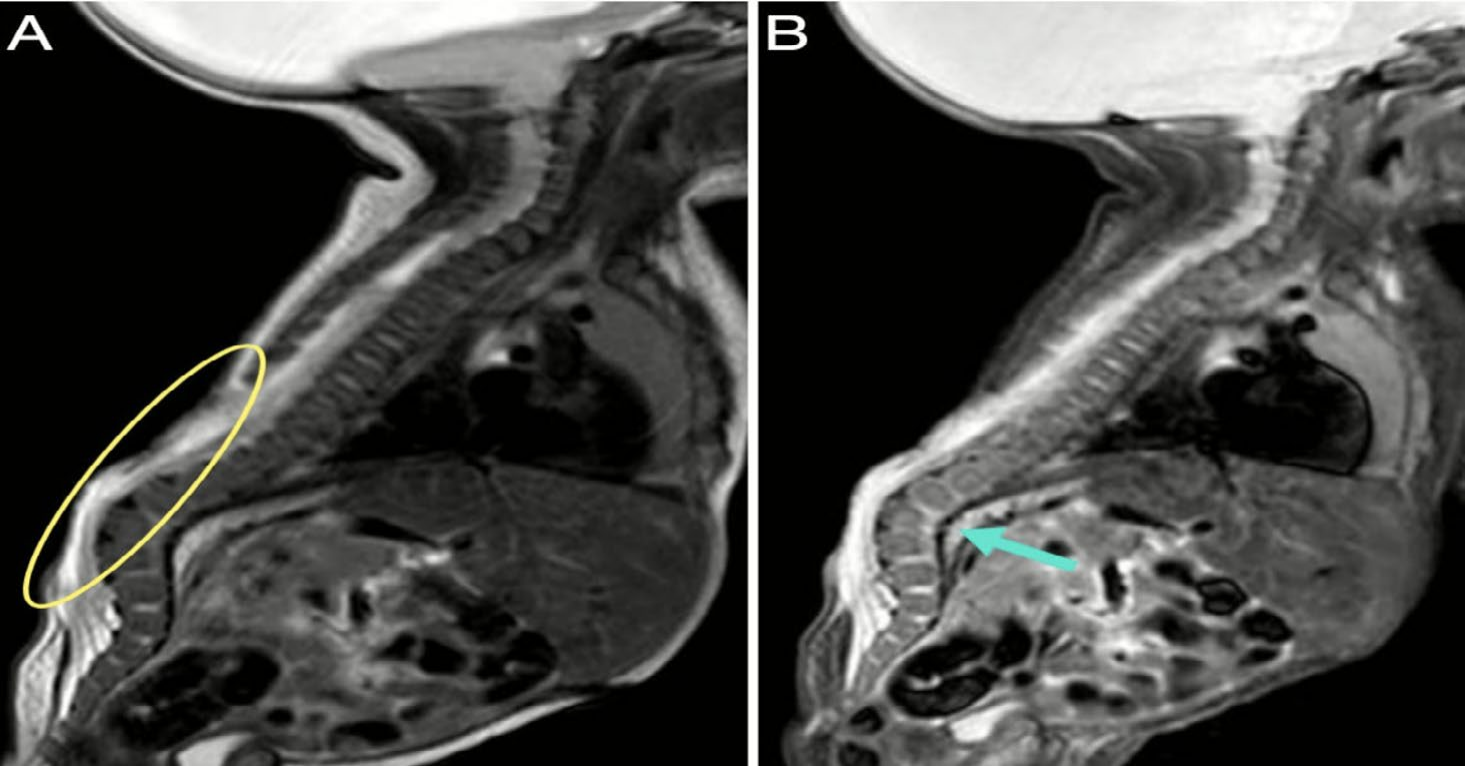
Sagittal MRI of the spine—(A) T1‐weighted and (B) STIR sequences—illustrates multiple defects of the posterior vertebral arches across the lower thoracic, lumbar, and sacral regions, in keeping with spina bifida occulta (yellow oval). A pronounced lumbar kyphosis is also evident (blue arrow). Preoperative images are unavailable.

**FIGURE 3:**
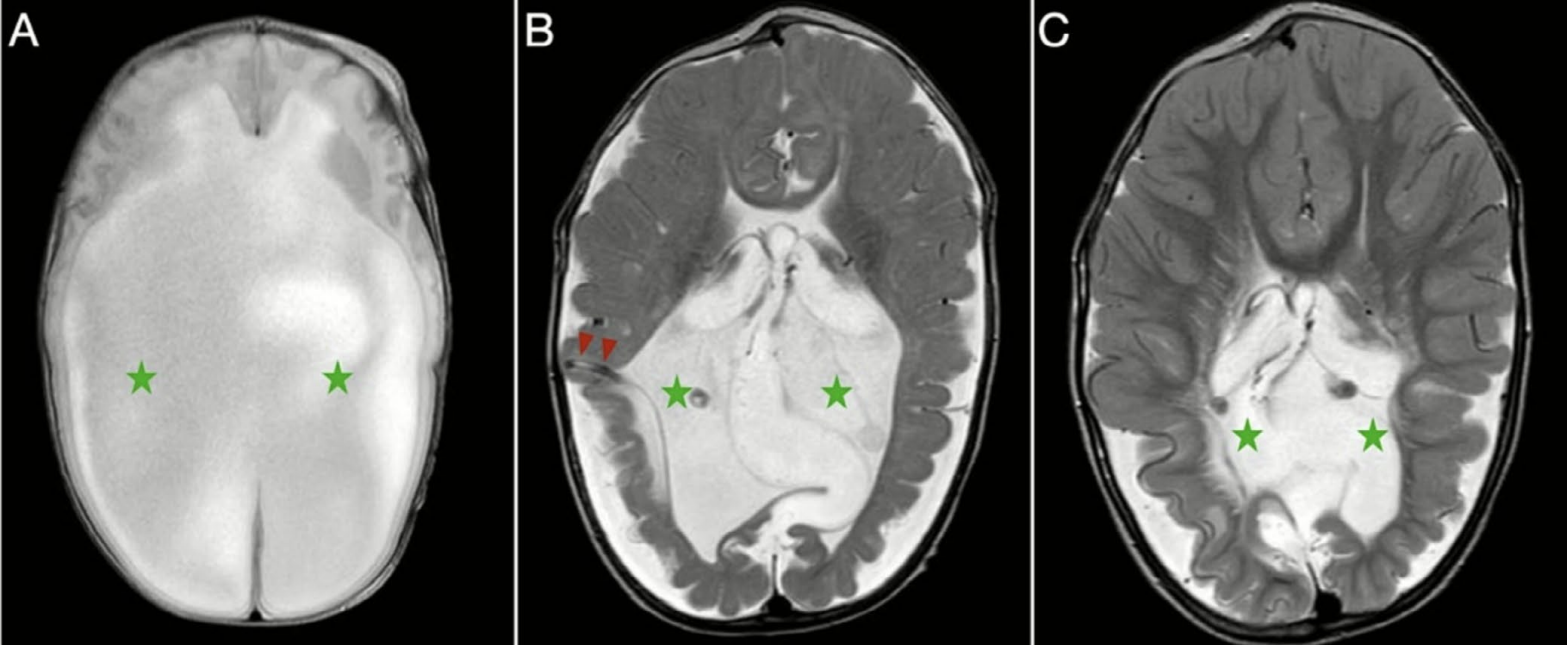
Serial axial T2‐weighted MRI images of the brain—(A) at presentation, (B) following insertion of a ventriculoperitoneal (VP) shunt (burgundy arrowheads), and (C) on two‐year follow‐up—demonstrate progressive reduction of hydrocephalus (green stars).

Surgical closure of the myelomeningocele was performed on the patient 2 days after birth (Figure 2), and a VP shunt was inserted 12 days later (Figure 3).

The patient did not experience any complications during the postoperative period. He remained hemodynamically stable, tolerated feeds well, and there were no new neurological deficits. He is still being closely monitored by a multidisciplinary team in coordination with neurosurgery and pediatrics for ongoing assessment of ventricular size and neurodevelopmental progress.

**FIGURE 4:**
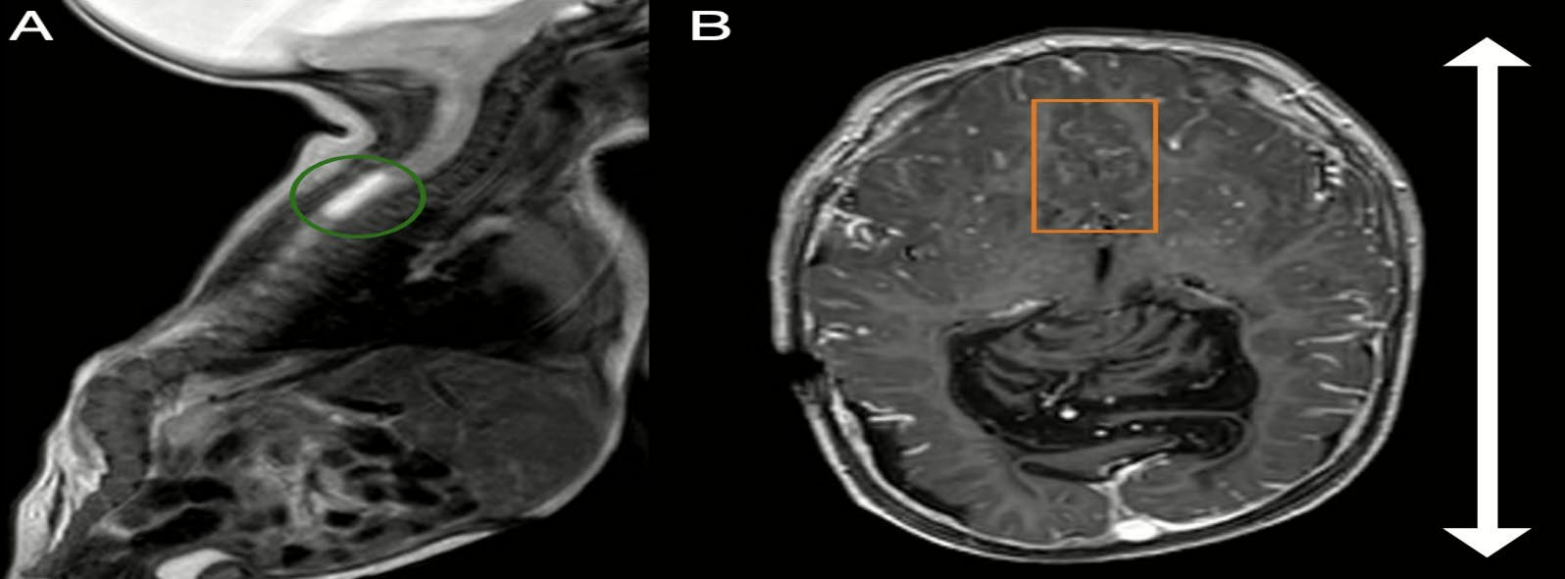
(A) Selected sagittal T2‐weighted MRI image of the spine demonstrates a dilated central canal extending from approximately D1 to D3 vertebral levels, consistent with a small syrinx (green circle). (B) Selected axial contrast‐enhanced T1‐weighted MRI image of the brain demonstrates a fenestrated falx cerebri, manifested by interdigitating gyri within the midline defect (orange rectangle). Note the increased anteroposterior (AP) diameter of the skull, indicated by a bidirectional double‐headed white arrow, suggesting scaphocephaly.

MRI of the brain and spine showed several significant findings. The spinal cord contained a small syrinx (Figure 4A). The skull showed scaphocephaly (Figure 4B).

**FIGURE 5:**
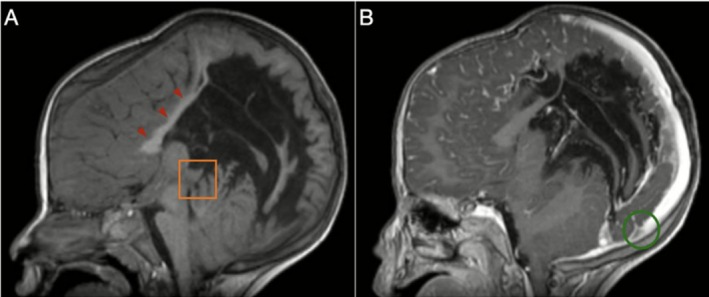
(A) Pre‐ and (B) post‐contrast sagittal T1‐weighted MRI images of the brain demonstrate dysgenesis of the corpus callosum (red arrowheads), predominantly involving its posterior segment, beaking of the tectal plate (orange square), and a low‐lying torcula (green circle).

In addition, the septum pellucidum was missing. There was also a full‐blown corpus callosum dysgenesis (Figure 5).

**FIGURE 6:**
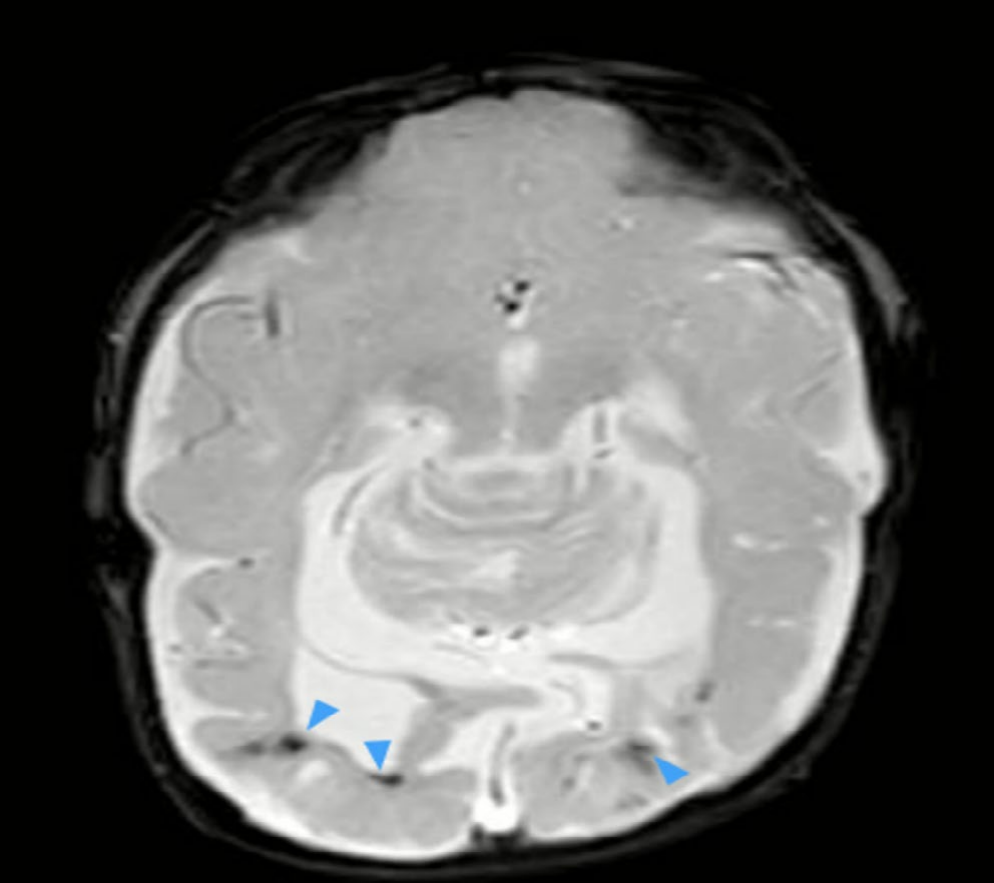
Axial SWI (T2‐weighted*) MRI image of the brain demonstrates a few intra‐ and extraventricular blooming artifacts (blue arrowheads), consistent with blood products.

In addition, the radiologist observed a few intra‐ and extraventricular blooming artifacts, which were considered suggestive of blood products (Figure 6).

## 4 Conclusion and Results

Arnold–Chiari Malformation Type II is a complex defect of neural development with serious neurological consequences. It is tightly associated with myelomeningocele and hydrocephalus. Radiology, especially MRI, is still the cornerstone to determine the extent of herniation and to discover any related anomaly in the brain and spine. In this case, imaging was important for both the diagnosis and the surgical plan, which contributed to favorable clinical outcomes. On the other hand, prevention through maternal folic acid intake before pregnancy is still very crucial in curbing the occurrence of neural tube defects. In the end, if the infant is diagnosed early, repaired surgically on time, and given the necessary multidisciplinary care, the chances of survival and good development are significantly increased.

## 5 Discussion

### 5.1 Overview Features

Chiari Malformation, a condition first recognized by Hans Chiari in 1891, describes a group of congenital hindbrain malformations that are the result of defective neural tube closure and abnormal CSF circulation during early embryonic development [1]. Its pathogenesis is multifactorial and involves both genetic and environmental factors, such as folate deficiency, maternal diabetes, and exposure to teratogens [6], which are particularly relevant in our patient given the absence of periconceptional folic acid supplementation.

Four types of Chiari Malformation are distinguished today, each type bearing its own characteristic anatomical features [9]. The cerebellar tonsils of Type I are descended caudally by more than 5 mm below the foramen magnum, and this is often accompanied by syringomyelia [10]. Type II, also referred to as Arnold–Chiari Malformation, is the most typical and most intricate version, which involves the downward movement of cerebellar tonsils, vermis, medulla, and fourth ventricle through the foramen magnum [11]. It is almost always coupled with myelomeningocele and hydrocephalus because of posterior fossa crowding and CSF flow blockage [11], as clearly demonstrated in our patient. Type III is depicted as a high cervical or low occipital encephalocele with cerebellar tissue inside [12], whereas Type IV is marked by cerebellar hypoplasia or aplasia [13].

### 5.2 Clinical Associations and Complications

The clinical features of Chiari II Malformation (CM‐II) depend on the extent of hindbrain herniation and the associated spinal and cranial defects [1]. Among the affected, around 20% of children show clinical signs such as poor feeding, neurogenic dysphagia, stridor, respiratory distress, and paresis of the lower limbs [14], with motor deficits being evident in our patient in the form of complete lower limb paralysis. Vocal cord paralysis, which occurs in about 30% of patients, can cause phonation changes, and the respiratory drive being impaired may cause recurrent aspiration and apnea [14].

Hydrocephalus is usually non‐communicating and occurs as a consequence of the blockage of the CSF routes at the regions of the fourth ventricle or aqueduct of Sylvius [15], consistent with the severe ventricular dilatation observed in our case. Myelomeningocele is the anomaly associated most frequently, and it causes both motor and sensory impairment beneath the lesion level; this often results in a neurogenic bladder and varying levels of paralysis [16]. Among other frequently observed anomalies, the most common intracranial findings include corpus callosum dysgenesis, absence of the septum pellucidum, and cranial vault abnormalities such as craniolacunia or scaphocephaly [17]. Notably, our patient demonstrated several of these associations, including corpus callosum dysgenesis, absence of the septum pellucidum, scaphocephaly, and syringomyelia. The overall prognosis is to a great extent determined by the degree of neurological involvement and the presence of such associated malformations [2].

### 5.3 Diagnostic Approaches

Prenatal imaging progress has led to a drastic enhancement in the early identification of CM‐II and related defects in the neural tube [18]. Ultrasonography remains the primary screening modality, where classic findings include the “lemon sign” and “banana sign” which are indicative of neural tube defects and hindbrain herniation, respectively [14]. Maternal serum alpha‐fetoprotein testing is another effective method for prenatal detection [19]. After birth, cranial ultrasound through the fontanelle is an easily performed bedside procedure for hydrocephalus and ventricular dilation detection without the risk of radiation exposure [20]. Nevertheless, MRI is the definitive and main method of evaluation. MRI not only provides excellent soft tissue contrast but also gives the precise details of the posterior fossa structures, the extent of cerebellar and brainstem herniation, the size of the ventricle, and the presence of spinal cord lesions such as syringomyelia [14].

### 5.4 Treatment Outcomes

CM‐II management is a collaboration of specialists from various fields including pediatric neurosurgery, neonatology, and physiotherapy. The first step is to perform a surgical procedure after the birth, which is myelomeningocele closure; this act of surgery has the benefit of preventing infection and at the same time maintaining the integrity of the neural tissues [21], as was successfully performed in our patient within the first 48 h of life. The insertion of a ventriculoperitoneal (VP) shunt is a common procedure that is performed for the treatment of hydrocephalus and is typically performed during the first 2 weeks of life [22], which resulted in marked radiological improvement and clinical stability in our case.

This case highlights the importance of early diagnosis, prompt surgical intervention, and coordinated multidisciplinary care in optimizing both survival and long‐term neurodevelopmental outcomes in patients with Chiari II Malformation.

## Author Contributions

**Mohammad Alashqar:** software, supervision, writing – original draft, writing – review and editing. **Seba Lubbadeh:** software, supervision, writing – original draft, writing – review and editing. **Ahmad Daraghmeh:** writing – original draft, writing – review and editing. **Zaina Khaled:** writing – original draft, writing – review and editing. **Ahmad Alashqar:** writing – original draft, writing – review and editing. **Suleiman Sbeih:** writing – original draft, writing – review and editing. **Amjad Bdair:** writing – original draft, writing – review and editing. **Israa Salman:** writing – original draft, writing – review and editing. **Mohammed I. Abu Kamesh:** writing – original draft, writing – review and editing.

## Funding

The authors have nothing to report.

## Ethics Statement

Informed consent was obtained from the patient's parents. Our institution does not require ethical approval for case reports.

## Consent

Written informed consent was obtained from the patient's parents for publication of this case report and the accompanying images.

## Conflicts of Interest

The authors declare no conflicts of interest.

## Data Availability

The data supporting the findings of this case report is not available due to privacy and confidentiality restrictions.
